# Factors affecting the mental health of the LGBTQ+ population in Greece

**DOI:** 10.3389/fpsyg.2026.1811328

**Published:** 2026-05-15

**Authors:** Anastasios Charalampakis, Stergios Kaprinis, Agorastos Agorastos, Eleni Parlapani

**Affiliations:** Department of Psychiatry, School of Medicine, Faculty of Health Sciences, Aristotle University of Thessaloniki, Thessaloniki, Greece

**Keywords:** depression, LGBTQ+ mental health, minority stress, perceived stress, structural stigma, suicidality

## Abstract

**Objective:**

To examine associations between general stressors and minority stressors (including structural stigma and rejection sensitivity), personality traits, and mental health outcomes (perceived stress, psychological distress, depressive symptoms, and suicidality) among LGBTQ+ and non-LGBTQ+ adults in Greece, integrating minority stress and temperament/trait frameworks.

**Methods:**

A cross-sectional, non-probabilistic survey of 389 adults in Greece (63% LGBTQ+) was conducted between January and December 2024 using a self-administered questionnaire distributed primarily online, with a parallel paper version entered into the same electronic database using standardized procedures. Measurement quality was supported by confirmatory factor analyses, reliability indices, and differential item functioning tests. Group comparisons and multivariable linear regression models, including interaction terms, were used to examine associations with outcomes.

**Results:**

LGBTQ+ participants reported higher perceived stress, psychological distress, and depressive symptoms than non-LGBTQ+ participants. In the overall sample, extraversion, agreeableness, and conscientiousness were associated with lower stress-related outcomes; however, these associations were weaker among LGBTQ+ participants, and conscientiousness was positively associated with stress in gender-expansive participants. Structural stigma was associated with greater distress, with patterns varying across subgroups. Higher educational attainment was inversely associated with depressive symptoms, particularly among LGBTQ+ participants. Suicidality was most strongly associated with depressive symptoms, while the association between psychological distress and suicidality appeared weaker among LGBTQ+ participants.

**Conclusion:**

Minority stress indicators, structural stigma, and personality traits showed distinct patterns of association with mental health outcomes in this Greek sample, with evidence that minority stress conditions may attenuate typical trait-related buffering. Within the constraints of the cross-sectional design, the findings support stigma-informed public mental health interventions and policies that reduce structural barriers and strengthen context-appropriate protective resources for sexual and gender minorities.

## 1 Introduction

Mental health disparities among LGBTQ+ individuals are well documented. Minority Stress Theory, developed by Meyer (2003, 1995), proposes that chronic exposure to stigma, prejudice, and discrimination generates psychological strain, increasing vulnerability to depression, anxiety, and other mental health disorders. These stressors operate at multiple levels: distal factors, such as victimization and discrimination, and proximal factors, including internalized stigma and expectations of rejection (Meyer, 2003; Meyer, 1995; Meyer and Frost, 2013; Hoy-Ellis, 2021; Meyer, 2015). Importantly, these processes affect both sexual and gender minorities and interact with broader social determinants of health, with mechanisms including coping strategies and social support deficits (Meyer, 2015; Hoy-Ellis, 2021).

Building on this framework, the Psychological Mediation Framework emphasizes cognitive, emotional, and social mechanisms that mediate the impact of stigma-related stress on psychopathology. Emotional dysregulation, cognitive biases, and lack of social support are key mediators, while structural stigma—systemic barriers limiting opportunity and well-being—can amplify negative outcomes (Hatzenbuehler, 2009; Hatzenbuehler and Link, 2014; Hatzenbuehler et al., 2024). Complementing these models, the Rejection Sensitivity Model, adapted for LGBTQ+ populations, posits that repeated experiences of rejection increase vigilance toward future rejection, perpetuating cycles of anxiety and interpersonal difficulties (Feinstein, 2019; Feinstein et al., 2012; Wells et al., 2019; Slimowicz et al., 2020; Feinstein et al., 2022; Xu et al., 2022; Hollinsaid et al., 2023).

Temperament Theory further suggests that biological and genetic predispositions interact with minority stress, heightening vulnerability (Bailey, 2019; Zucker et al., 2016; Allen and Robson, 2020; Junkins et al., 2024). Additionally, Cognitive Theory highlights the role of negative schemas, shaped by early experiences of stigma, in sustaining maladaptive emotions and behaviors (Beck and Haigh, 2014; Beck, 2010; Pachankis et al., 2015; Yolaç and Meriç, 2020a; Atasever, 2023). Together, these frameworks offer a comprehensive understanding of the psychological processes underlying mental health disparities in LGBTQ+ populations.

Epidemiological research demonstrates that LGBTQ+ individuals experience higher rates of mental health disorders than their heterosexual and cisgender peers. In the United Kingdom, over half of LGBTQ+ respondents reported depression, and 46% of transgender participants reported suicidal ideation (LGBT in Britain, 2018). U.S. studies indicate elevated depression and anxiety across all adult age groups, with the highest prevalence among young adults (18–29 years) (Marlay et al., 2022; MHA, 2025). In Southeast Asia, depression rates range from 23.5 to 40.3%, suicidal ideation reaches up to 58.9%, and suicide attempts range between 13.1 and 35.1% (Tan and Saw, 2022). Similar trends have been observed in Australia, Brazil, Ireland, and Greece (ABS, 2023; De Mattos et al., 2021; Higgins et al., 2024; ELSTAT, 2020).

The Minority Stress Model (MSM) links distal and proximal stressors to adverse mental health outcomes, including depression, anxiety, and suicidality (Bauerband et al., 2018; Shangani et al., 2019; DiGiuseppi et al., 2021; Kittiteerasack et al., 2020). However, critiques note its limited attention to intersectionality, cumulative stress, cultural variability, and protective factors such as social support, resilience, and positive identity (Chang et al., 2022; Kaufman et al., 2022). The Temporal Intersectional Minority Stress (TIMS) model extends MSM by incorporating developmental, historical, and generational influences (Botelho et al., 2023).

Empirical studies reinforce MSM’s explanatory power. In the U.S., transgender individuals report higher discrimination and vigilance than cisgender peers (Sun et al., 2020), while multiple minority identities combined with lower socioeconomic status are associated with elevated stress (Shangani et al., 2019; Mann et al., 2022). Evidence indicates that racial discrimination is linked to early-stage depression, whereas internalized homophobia and sexuality-related discrimination influence later-stage outcomes (DiGiuseppi et al., 2021; Fedele et al., 2022). Cross-cultural studies highlight the protective role of family support, resilience, and coping strategies, while victimization, harassment, and internalized stigma exacerbate mental health risk (Hinds et al., 2024; Liang and Huang, 2021; Thoma et al., 2021; Koziara et al., 2022; Wilson et al., 2023; Camposano et al., 2024; Hui and Berezina, 2024; Borgogna and Aita, 2021; Logie et al., 2020). Protective factors such as age, social support, and positive identity consistently mitigate adverse outcomes across diverse contexts (Zhou et al., 2024; Wang et al., 2020a; Mahon et al., 2021; Lee et al., 2022; Pellicane et al., 2023).

The Psychological Mediation Framework underscores the role of cognitive, emotional, and social mediators. Research in Jamaica, China, Ireland, Taiwan, and the U.S. shows that coping strategies, social support, and cognitive restructuring can reduce depression and anxiety, with gender and sexual orientation moderating these effects (Logie et al., 2020; Zhou et al., 2024; Wang et al., 2020b; Mahon et al., 2021; Lee et al., 2022; Wells et al., 2019; Slimowicz et al., 2020; Feinstein et al., 2022; Xu et al., 2022; Hollinsaid et al., 2023). Similarly, heightened vigilance to potential rejection increases vulnerability to depressive and anxiety symptoms, while internalized stigma and discrimination are associated with day-to-day mood fluctuations (Feinstein, 2019; Feinstein et al., 2012). Temperament Theory emphasizes the moderating role of personality traits, particularly among bisexual and pansexual individuals, in shaping stress responses (Bailey, 2019; Zucker et al., 2016; Allen and Robson, 2020; Junkins et al., 2024). Cognitive theories, drawing on Beck, demonstrate that cognitive distortions and internalized stigma are associated with depressive symptoms across contexts, as shown in Cyprus, Turkey, Brazil, and Romania (Beck and Haigh, 2014; Beck, 2010; Pachankis et al., 2015; Yolaç and Meriç, 2020b; Atasever, 2023; Silva et al., 2024; Popa-Velea et al., 2019).

Psychosocial risk factors shared with non-LGBTQ+ populations, such as abuse, low self-esteem, impulsivity, and limited social support, are compounded by intersectional identities (Clements-Nolle et al., 2018; Liao et al., 2015; Rosario et al., 2005; Platt and Scheitle, 2018; Wu et al., 2023; Alibudbud, 2022). Social support consistently buffers mental health outcomes, with family, peers, and LGBTQ+ community networks offering protective effects (Harper et al., 2022; Pitonak et al., 2020; Källström et al., 2022; Janković et al., 2020; Rogowska and Cisek, 2024; Wang et al., 2020c; Kaniuka et al., 2019). Religious and spiritual contexts may exert mixed effects: supportive environments enhance well-being, whereas rigid or rejecting settings increase psychological distress (Boppana and Gross, 2019; Kim et al., 2024; Exline et al., 2021; Wilson et al., 2021; Mahjoubi et al., 2024a). Structural vulnerabilities—including incarceration, homelessness, socioeconomic disadvantage, migration status, and chronic illness—interact with minority stress to further exacerbate mental health risk, consistent with biopsychosocial and syndemic models (Brandtzæg Godø et al., 2024; Timmins et al., 2019; Godfrey et al., 2023; Charak et al., 2023; Rogers et al., 2021; Ibrahim et al., 2022; Li et al., 2024; Wang et al., 2020a; Liang and Chen, 2024; Kelly et al., 2021; Stanton et al., 2025; Liu et al., 2021; López et al., 2022; Thomas et al., 2022; McGuire et al., 2024; DiGuiseppi et al., 2022; Shepherd et al., 2025). Gender, conceptualized as the dynamic interplay of body, identity, and social role, intersects with other identities and structural factors to shape mental health trajectories (Hall et al., 2023; Clark et al., 2021b).

Recent studies highlight unique vulnerabilities among LGBTQ+ migrants, adolescents, and refugees. Migrant populations experience elevated depression, anxiety, and suicidality due to compounded minority stress and acculturation challenges (Clark et al., 2021a; Harkness et al., 2021; Chaoudhry et al., 2025). Similarly, LGBTQ+ adolescents face intersectional risks associated with family rejection, school victimization, and identity development (Standley and Foster-Fishman, 2021; Donaldson et al., 2023; McPherson et al., 2023; Khanolkar et al., 2023).

Building on this extensive literature, the present study aims to examine stressors affecting the mental health of LGBTQ+ individuals in Greece, focusing on psychological distress, depression, and suicidality. Both general and minority-specific stressors were assessed, alongside personality traits informed by Temperament Theory. The study draws on insights from Kaprinis and Charalambakis (2026), whose pilot research guided the selection of instruments and confirmed the factorial structures used in this investigation.

## 2 Methods

### 2.1 Data collection procedure, pilot and Main study

The study used a self-administered questionnaire distributed electronically via Google Forms, complemented by a paper version of the same instrument. Most participants completed the questionnaire online, while a subset was recruited in person in a clinical setting, where the printed questionnaire was completed and later entered verbatim into the electronic database by the researcher and trained collaborators using standardized procedures. This process ensured a unified dataset, consistent variable coding, and reduced the risk of data-entry errors.

Data collection for the main study spanned from January to December 2024. Within this period, a pilot phase was conducted from June to September 2024 to assess question comprehension, estimated completion time, questionnaire acceptability and functionality, as well as the feasibility of recruitment through multiple channels. The results of the pilot phase, reported in Kaprinis and Charalambakis (2026), directly informed the final selection of research instruments, minor item revisions, and the factorial structures adopted in the main study.

### 2.2 Distribution channels and sampling strategy

Sampling was non-probabilistic, combining convenience sampling, community-based recruitment, and snowball methods, whereby participants shared the invitation within their social networks. Online recruitment occurred through LGBTQ+ groups on Facebook (with prior approval from administrators or moderators), networking via the NGO “Positive Voice” with member outreach conducted upon permission, a public information page disseminated through social media, and university community forums.

In-person distribution took place in a clinical setting at the outpatient clinics of the 2nd University Psychiatric Department of Aristotle University of Thessaloniki. Completed paper questionnaires were checked for completeness and entered into the electronic form using standardized procedures, including uniform entry, field verification, and random cross-checks.

### 2.3 Inclusion and exclusion criteria

Participants were eligible if they were 18 years or older and provided informed consent; only one participation per individual was allowed. Participation was voluntary. Exclusion criteria included lack of informed consent, failure to meet basic eligibility criteria, evidence of multiple entries, and extremely incomplete responses.

For the purposes of the present study, extremely incomplete responses were defined as those with >30% missing data or missing key outcome variables, which did not contain sufficient information to support the planned analyses. Respondents could withdraw at any time without consequence. To protect vulnerable participants, support resources and contact information were provided at the end of the form for anyone experiencing distress.

### 2.4 Questionnaire modifications based on pilot study

The pilot study informed several modifications: neutral demographic questions were placed first to establish trust, and transitional statements were added to clarify reference periods (e.g., “during the past two weeks”). Repetitive items were removed, and questions on gender identity and sexual orientation were revised using inclusive, non-pathologizing language. The income item was converted from an open-ended numeric question to an ordinal categorical variable to improve data quality and participant comfort. After these adjustments, the final version was stabilized for the main data collection phase.

### 2.5 Research instruments

The variables of interest were assessed using instruments whose factorial structures were established in the pilot study (Kaprinis and Charalambakis, 2026).

#### 2.5.1 Perceived stress

Measured using the PSS-14 (Cohen et al., 1983). The Greek version (Katsarou et al., 2012) has been validated for LGBTQ+ populations, and the pilot study indicated a two-factor structure reflecting self-efficacy and hopelessness.

#### 2.5.2 Psychological distress

Assessed with the Kessler-6 (K-6) (Kessler et al., 2002). The pilot study suggested a two-factor structure (anxiety and depression), consistent with international literature supporting its use in LGBTQ+ populations.

#### 2.5.3 Depression

Measured using the Beck Depression Inventory (BDI) (Beck, 1961). The Greek version (Ntonia and Demertzi, 1983) was supported by the pilot study through a two-factor solution (emotional-cognitive and somatic symptoms).

#### 2.5.4 Suicidality

Assessed using the Fountoulakis Risk Assessment of Suicidality Scale (RASS) (Fountoulakis et al., 2012). The pilot study confirmed a ten-item, one-factor structure for this Greek instrument.

#### 2.5.5 Rejection sensitivity

Measured using the Berenson et al. (2009) scale. Scores were computed as the product of anxiety × expected rejection, with the pilot study supporting a two-factor structure.

#### 2.5.6 Structural stigma

Measured with a 4-item scale (Kaprinis and Charalambakis, 2026), informed by structural stigma theory. Pilot analyses revealed two factors: personal experience and perceived societal attitudes.

#### 2.5.7 Internalized LGBTQ+-phobia

Assessed using the IHS Scale (Ross and Rosser, 1996). The pilot study supported the 23-item, three-factor solution: public identification, internalized stigma, and social pressure.

#### 2.5.8 Personality traits

Assessed using the NEO-FFI (Rammstedt and John, 2007). This instrument was selected over the BFI-10 due to superior reliability and validity demonstrated in the pilot phase.

#### 2.5.9 Traumatic exposure

Measured using the Stressful Life Events Questionnaire (Goodman et al., 1998), with a one-factor solution relevant for LGBTQ+-specific events.

### 2.6 Ethics approval

Informed Consent: The patients/participants provided their written informed consent to participate in this study.

The study was approved by the Bioethics and Ethics Committee of the School of Medicine, Aristotle University of Thessaloniki (Protocol Number: 175/2024). No direct identifiers were collected, ensuring participant confidentiality and anonymity.

## 3 Results

### 3.1 Socio-demographic characteristics

The study sample consisted of 389 participants, of whom 63.0% identified as LGBTQ+. The age distribution was as follows: 18–25 years (15.2%), 26–35 years (20.8%), 36–45 years (39.3%), 46–65 years (21.8%), and ≥66 years (2.8%). Educational attainment was predominantly tertiary (45.5%) or postgraduate (25.4%), with smaller proportions having completed high school (12.6%), post-secondary education (7.2%), lower secondary or primary education (5.4%), or doctoral degrees (3.9%).

Employment status included private-sector employment (31.2%), self-employment (30.7%), and public-sector employment (16.5%). Marital status was distributed among those in a relationship or cohabiting (39.3%), married or in a civil partnership (29.6%), single (28.8%), and divorced or widowed (2.3%). Most participants (73.1%) had no children. Household income categories were €0–800 (24.2%), €801–1,500 (30.3%), €1,501–2,500 (27.5%), €2,501–3,500 (14.7%), and >€3,500 (3.3%).

Most participants were Greek nationals (83.8%) residing in Attica (53.5%), Crete (18.5%), or Central Macedonia (9.0%). Religious affiliation was primarily Christian (65.2%), followed by atheist (22.4%) and agnostic (9.0%). Most reported no chronic illness (69.4%) and no substance use (82.8%). Gender identity included cisgender men (56.8%), cisgender women (31.1%), transgender men (5.4%), and non-binary/queer individuals (5.7%).

### 3.2 Scale validity and reliability

As suggested by the pilot findings, Table 1. summarizes the reliability and factor structure for each instrument in the main study sample.

**Table 1:** Summary of reliability, CFA, and DIF results.

Measure	*α*	*ω*	CFI	TLI	RMSEA	SRMR	DIF
PSS-14	0.88	0.88	0.98	0.96	0.06	0.05	Minimal
K-6	0.91	0.92	1.00	1.00	0.00	0.01	None
BDI	0.96	0.96	0.96	0.94	0.07	0.04	Minimal
RASS	0.88	0.90	0.97	0.94	0.09	0.05	Minor
SS	0.63	0.68	1.00	1.00	0.02	0.01	Group differences
RSS	0.93	0.93	0.97	0.96	0.07	0.06	Minimal
SLESQ	0.72	0.73	0.97	0.96	0.04	0.04	Several
IHS	0.88	0.89	0.99	0.96	0.05	0.04	Moderate
NEO-FFI	0.77–0.88	0.79–0.88	0.90	0.75	0.07	0.12	Moderate

### 3.3 Psychometric properties by instrument

- Perceived Stress Scale (PSS-14): CFA supported both two-factor (helplessness and self-efficacy) and one-factor structures, with very good internal consistency (total *α* = 0.882). DIF analysis revealed minimal bias for LGBTQ+ participants.
- Kessler Psychological Distress Scale (K-6): Supported both one- and two-factor (anxiety and depression) models with excellent fit and reliability (total *α* = 0.910). No significant DIF was found.
- Beck Depression Inventory (BDI): CFA supported one- and two-factor models (cognitive–affective and somatic). Reliability was excellent (total *α* = 0.956).
- Risk Assessment of Suicidality Scale (RASS): CFA favored a three-factor structure (life, intention, history). Reliability was good (*α* = 0.880), though the “history” factor showed lower consistency (*α* = 0.389).
- Structural Stigma Scale (SS): Supported a two-factor model (personal experience and social attitudes) with excellent fit. DIF revealed higher personal experience but lower perceived societal support among LGBTQ+ participants.
- Rejection Sensitivity Scale (RSS): Supported two factors (anxiety and expectation) with excellent reliability (*α* > 0.91).
- Internalized LGBTQ+-phobia Scale (IHS): CFA supported a three-factor model (public identification, social pressure, internal stigma) with high reliability (*α* = 0.73–0.88). DIF was noted between gender-expansive and cisgender LGBTQ+ participants.
- Personality Traits (NEO-FFI): Supported the five-factor structure with moderate fit and good reliability (*α* = 0.77–0.88).

Concurrent validity was supported by theoretically consistent Pearson correlations. Perceived stress was strongly associated with psychological distress (*r* = 0.863), depression (*r* = 0.807), and neuroticism (*r* = 0.787), while negatively associated with extraversion (*r* = −0.549) and conscientiousness (*r* = −0.262).

### 3.4 Descriptive statistics

Descriptive analyses of key variables (*N* = 389) are presented in Table 2. Participants reported a mean level of perceived stress of 29.53 (SD = 9.47) and psychological distress of 12.01 (SD = 5.91). Depression scores (*M* = 15.46, SD = 14.29) showed wide variability, indicating the presence of individuals with high symptomatology.

**Table 2:** Descriptive statistics for the research variables (*N* = 389).

Variable	Mean	Median	SD	Min	Max	5th	25th	75th	95th
Perceived stress	29.53	29.00	9.47	4.00	52.00	16.00	21.00	37.00	45.00
Psychological distress	12.01	12.00	5.91	0.00	24.00	3.00	6.00	18.00	20.00
Depression (BDI)	15.46	11.00	14.29	0.00	51.00	0.00	3.00	27.00	45.00
Structural stigma	11.26	11.00	3.01	4.00	20.00	6.00	9.00	13.00	16.00
Suicidality	6.93	5.00	5.78	1.00	30.00	1.00	1.00	11.00	17.00
Rejection sensitivity	12.72	10.44	8.22	1.00	29.10	2.78	6.44	20.56	27.33
Stressful life events	3.39	3.00	2.61	0.00	13.00	0.00	1.00	5.00	8.00
Public ID (LGBTQ+)*	31.62	32.00	7.93	13.00	45.00	20.00	25.00	38.00	45.00
Internalized stigma*	27.05	27.00	9.49	10.00	42.00	12.20	19.00	34.00	41.00
Social oppression*	16.33	16.00	3.29	6.00	20.00	10.00	14.00	20.00	20.00
Neuroticism	35.91	36.00	9.81	12.00	59.00	22.40	27.00	43.00	53.00
Extraversion	36.96	38.00	7.15	19.00	55.00	25.00	30.00	42.00	48.00
Openness	40.10	40.00	6.30	16.00	54.00	32.00	36.00	46.00	49.00
Agreeableness	39.35	40.00	8.12	23.00	59.00	28.00	31.00	46.00	52.60
Conscientiousness	42.67	42.00	6.20	26.00	56.00	33.40	38.00	47.00	53.00
*N = 245 for LGBTQ+-specific variables									

### 3.5 Factors affecting mental health

Multiple linear regression models were estimated to identify predictors of mental health outcomes. Given the complexity and number of interaction terms, these results represent associative patterns requiring cautious interpretation (Tables 3–10).

**Table 3:** Omnibus ANOVA for predictors of perceived stress—total sample.

Predictor	Sum of squares	df	Mean square	*F*	*p*
Income	323.66	4	80.91	4.413	0.002
Region	428.02	11	38.91	2.122	0.019
Religion	181.15	4	45.29	2.470	0.045
Extraversion	220.96	1	220.96	12.051	<0.001
Agreeableness	77.66	1	77.66	4.236	0.041
Extraversion * LGBTQ	98.72	1	98.72	5.385	0.021
Religion * LGBTQ	290.39	4	72.60	3.960	0.004
RSS * LGBTQ	81.85	1	81.85	4.464	0.036

**Table 4:** Omnibus ANOVA for predictors of perceived stress—LGBTQ sample.

Predictor	Sum of squares	df	Mean square	*F*	*p*
Marital status	175.050	3	58.350	3.586	0.015
Religion	334.751	6	55.792	3.429	0.003
Chronic illness	177.599	1	177.599	10.915	0.001
Neuroticism	575.979	1	575.979	35.399	<0.001
Conscientiousness	334.828	1	334.828	20.578	<0.001
Conscientiousness * GE	105.517	1	105.517	6.485	0.012

**Table 5:** Omnibus ANOVA for predictors of psychological distress—total sample.

Predictor	Sum of squares	df	Mean square	*F*	*p*
Perceived stress (PSS)	162.340	1	162.340	28.239	<0.001
Life events (SLE)	46.011	1	46.011	8.003	0.005
Agreeableness	54.006	1	54.006	9.394	0.002
Religion	109.536	4	27.384	4.763	0.001
Absence of SS	53.100	1	53.100	9.237	0.003
SLE * LGBTQ	74.527	1	74.527	12.964	<0.001
Conscientiousness * LGBTQ	49.234	1	49.234	8.564	0.004
LGBTQ * Absence of SS	78.538	1	78.538	13.662	<0.001

**Table 6:** Omnibus ANOVA for predictors of psychological distress—LGBTQ Sample.

Predictor	Sum of squares	df	Mean square	*F*	*p*
Income	60.5780	4	15.1445	2.597	0.038
Absence of SS	46.5095	1	46.5095	7.975	0.005
Neuroticism	105.8932	1	105.8932	18.158	<0.001
Perceived stress	457.8139	1	457.8139	78.502	<0.001
Conscientiousness * GE	24.4075	1	24.4075	4.185	0.042

**Table 7:** Omnibus ANOVA for predictors of depression – total sample.

Predictor	Sum of squares	df	Mean square	*F*	*p*
Distress (KSS)	402.6066	1	402.6066	16.353	<0.001
Education	371.7657	5	74.3531	3.020	0.011
Income	241.7579	4	60.4395	2.455	0.046
Nationality	693.5988	9	77.0665	3.130	0.001
Neuroticism * LGBTQ	131.4438	1	131.4438	5.339	0.022
Education * LGBTQ	479.1057	5	95.8211	3.892	0.002

**Table 8:** Omnibus ANOVA for predictors of depression—LGBTQ sample.

Predictor	Sum of squares	df	Mean square	*F*	*p*
Occupation	1255.19	6	209.20	8.522	<0.001
Marital status	288.26	3	96.09	3.914	0.010
Income	309.24	4	77.31	3.149	0.016
Nationality	899.67	11	81.79	3.332	<0.001
Internal perception	342.52	1	342.52	13.953	<0.001
Neuroticism	290.69	1	290.69	11.841	<0.001
Distress (KSS)	1076.20	1	1076.20	43.840	<0.001
RSS * GE	185.46	1	185.46	7.555	0.007

**Table 9:** Omnibus ANOVA for predictors of suicidality—total sample.

Predictor	Sum of squares	df	Mean square	*F*	*p*
Depression (BDI)	103.878	1	103.878	11.167	<0.001
Distress (KSS) * LGBTQ	42.705	1	42.705	4.591	0.033

**Table 10:** Omnibus ANOVA for predictors of suicidality—LGBTQ sample.

Predictor	Sum of squares	df	Mean square	*F*	*p*
Age	152.864	5	30.573	2.862	0.017
Region	347.442	11	31.586	2.957	0.001
Religion	216.150	6	36.025	3.373	0.004
RSS	62.658	1	62.658	5.866	0.017
Depression	715.541	1	715.541	66.987	<0.001
Internal perception * GE	52.442	1	52.442	4.909	0.028
Neuroticism * GE	59.147	1	59.147	5.537	0.020
Openness * GE	52.700	1	52.700	4.934	0.028

### 3.6 Perceived stress

In the total sample, the model (Adj. R^2^ = 0.797) showed that higher extraversion (*β* = −0.465) and agreeableness (*β* = −0.178) were associated with lower stress. However, interactions indicated that extraversion’s protective effect was weaker among LGBTQ+ participants (*β* = 0.353, *p* = 0.021). Notably, higher income was associated with increased stress specifically within the LGBTQ+ group (*β* = 10.63, *p* = 0.024).

In the LGBTQ+ subsample (Adj. R^2^ = 0.806), higher neuroticism increased stress (*β* = 0.411), while conscientiousness was protective (*β* = −0.282). However, for gender-expansive (GE) participants, the interaction term (*β* = 105.52, *p* = 0.012) suggested a different relationship with conscientiousness.

### 3.7 Psychological distress

The total sample model (Adj. R^2^ = 0.836) identified perceived stress (*β* = 0.312) and stressful life events (SLE; *β* = 0.528) as positive predictors. Conscientiousness was protective in the general sample, but this effect was significantly weaker for LGBTQ+ individuals (*β* = 0.226, *p* = 0.004).

In the LGBTQ+ subsample (Adj. R^2^ = 0.811), structural stigma (*β* = 0.348), perceived stress (*β* = 0.413), and neuroticism (*β* = 0.194) were the strongest predictors of distress.

### 3.8 Depression

In the total sample (Adj. R^2^ = 0.880), higher psychological distress was the strongest correlate of depression (*β* = 0.924). Education showed a protective effect that was particularly strong for LGBTQ+ individuals. Unexpectedly, monthly income was positively associated with depression scores.

In the LGBTQ+ subsample (Adj. R^2^ = 0.885), occupation, income, and nationality were significant socio-demographic predictors. Internalized stigma (*β* = 0.39) and rejection sensitivity (*β* = 0.30) were significant psychological predictors.

### 3.9 Suicidality

For the total sample (Adj. R^2^ = 0.720), depression was the primary predictor of suicidality (*β* = 0.293). The association between general psychological distress and suicidality was found to be weaker among LGBTQ+ participants (*β* = −0.414, *p* = 0.033).

In the LGBTQ+ subsample (Adj. R^2^ = 0.777), suicidality was significantly associated with younger age (18–25), unemployment, and atheist/agnostic beliefs. Interactions with gender-expansiveness (GE) moderated the effects of internal stigma, neuroticism, and openness.

## 4 Discussion

This study demonstrates that mental health outcomes in Greece are shaped by a complex interplay of personality traits and stressors. A key finding is that minority stress appears to attenuate the typical protective effects of traits like extraversion and agreeableness. This research examined how these factors, along with socio-demographic variables, were associated with multiple domains of mental health—perceived stress, psychological distress, depressive symptoms, and suicidality—among LGBTQ+ and non-LGBTQ+ adults in Greece. Across outcomes, associations were heterogeneous, and in several models, LGBTQ+ status and gender expansiveness moderated trait- and stressor-outcome links, consistent with the possibility that minority stress conditions alter the usual pattern of associations between personality and distress.

Consistent with prior research on coping and personality, higher extraversion and agreeableness were linked with lower perceived stress in the overall sample. Among LGBTQ+ participants, however, the protective influence of these traits was weaker, suggesting that minority stress may blunt typical personality-related advantages. In addition, conscientiousness was unexpectedly associated with higher stress in gender-expansive individuals. This suggests a possible link to maladaptive over-control or perfectionism in the face of structural stigma, indicating that traits usually seen as adaptive might operate differently under minority stress conditions. Socio-demographic features such as income, geographical region, and religious affiliation also played a role. While greater income was associated with lower stress in the general population, it was associated with higher stress among LGBTQ+ participants, which could reflect unique pressures experienced by sexual and gender minorities in higher socioeconomic brackets.

Psychological distress was strongly associated with perceived stress, structural stigma, and neuroticism across all groups. Traits often considered protective—such as agreeableness and conscientiousness—appeared less beneficial for LGBTQ+ participants, reinforcing the finding that minority stress processes may attenuate personality-related resilience. Stressful life events increased distress in the full sample, but this relationship was somewhat weaker among LGBTQ+ individuals, possibly indicating differences in how stressors are perceived or managed within minority groups.

Depressive symptoms were primarily associated with higher psychological distress, greater neuroticism, and stronger internalized stigma. Educational attainment emerged as a consistent protective factor against depression, particularly for LGBTQ+ participants, which may reflect greater access to social resources or specialized coping skills. In contrast to expectations, higher income was linked with greater depression among LGBTQ+ participants. This interesting finding suggests that socioeconomic gains do not always translate into better mental health for minority individuals and may, at times, increase depression due to heightened visibility to prejudice or occupational minority stress. Rejection sensitivity and perceived social pressure were also pertinent contributors to depressive symptoms, especially in gender-expansive subgroups.

Suicidality showed its own pattern of predictors. Internalized stigma, psychological distress, and neuroticism were significant correlates, supporting theoretical links between minority stress and suicidal thoughts and behaviors. LGBTQ+ participants, in particular those who are gender-expansive, reported higher suicidality. Interactions with conscientiousness and openness suggest that these personality dimensions may affect suicidality risk differently depending on gender identity and minority status. This reinforces the view that suicidal tendencies should not be treated simply as extreme forms of depression or distress but as a distinct outcome shaped by psychosocial and identity-based factors.

Taken together, LGBTQ+ status was associated with increased vulnerability across outcomes, but the strength and nature of that vulnerability differed depending on the mental health domain and individual characteristics. Structural and internalized stigma consistently emerged as influential factors, yet their impacts were not uniform across subgroups. Gender-expansive individuals, in particular, showed distinct patterns of risk in relation to stress, distress, and suicidality, highlighting the importance of acknowledging diversity and the specific role of traits like conscientiousness within LGBTQ+ research.

### 4.1 Practical implications and future directions

These findings carry practical implications for clinical practice and policy. Mental health interventions should be tailored to account for both individual differences (such as personality and coping styles) and structural challenges (like stigma and exclusion). Approaches that enhance adaptive coping, reduce internalized stigma, and foster supportive environments may help improve outcomes. While education and socioeconomic resources may serve as resilience factors, their protective effects are not universal and must be considered within the specific context of minority stress.

Future research would benefit from longitudinal designs to clarify causal pathways, formal subgroup analyses within LGBTQ+ populations, and analytic approaches such as structural equation modelling. Such methods can test the interrelationships among minority stress, personality, and mental health outcomes more directly, further illuminating how structural barriers transform individual psychological traits.

### 4.2 Limitations

The study has limitations. First, the non-probabilistic sampling strategy (convenience, community-based recruitment, and snowballing) limits representativeness and may introduce selection bias, including potential overrepresentation of urban, younger, or more highly educated individuals and of persons connected to LGBTQ+ networks or clinical settings. Second, although online data collection can improve access to hard-to-reach populations, it relies on self-report and may be vulnerable to non-unique participation or careless responding; we mitigated these risks through eligibility restrictions (one participation per person), screening for multiple entries or indications of non-unique responses, and exclusion of extremely incomplete questionnaires. Third, the cross-sectional design precludes causal inference and limits conclusions about temporal ordering between minority stress, traits, and mental health outcomes. Fourth, some subgroup analyses - particularly for gender-expansive participants - may be underpowered, and findings should be interpreted cautiously and replicated in larger and more diverse samples. Longitudinal and probability-based studies, ideally combining survey and administrative data and incorporating objective indicators of structural stigma, are needed to clarify pathways and inform intervention timing, especially for suicidality.

## 5 Conclusion

In summary, mental health outcomes in this Greek sample were associated with a multifaceted interplay of personality, psychosocial stressors, minority stress indicators, and socio-demographic factors. Findings suggest that:

- Trait-Attenuation: Minority stress conditions are associated with the attenuation of the protective effects of personality traits.
- Regulatory Depletion: Mechanistically, structural stigma may deplete regulatory resources, while rejection sensitivity may induce hypervigilance.
- Maladaptive Adaptation: Internalized stigma may transform typically adaptive traits into maladaptive overcontrol.
- Socioeconomic Paradox: Higher income was associated with higher depression, possibly reflecting occupational minority stress or reporting bias.

LGBTQ+ individuals, and particularly gender-expansive persons, displayed distinct patterns of risk across outcomes, underscoring the need for intersectional and multilevel mental health strategies.

## Data Availability

The datasets generated and/or analyzed during the current study are not publicly available due to ethical and privacy restrictions concerning sensitive participant information, but may be made available by the corresponding author upon reasonable request and subject to applicable ethical and data protection requirements.
